# Research hotspots and trends of post-stroke depression rehabilitation: a bibliometric analysis from 2003 to 2024

**DOI:** 10.3389/fneur.2025.1526506

**Published:** 2025-04-04

**Authors:** Hongwei Cai, Shini Cai, Aihong Li, Aisong Guo

**Affiliations:** ^1^Department of Rehabilitation Medicine Center, Affiliated Hospital of Nantong University, Nantong, Jiangsu, China; ^2^School of Nursing and Rehabilitation, Nantong University, Nantong, Jiangsu, China; ^3^Department of Neurology, Affiliated Hospital of Nantong University, Nantong, Jiangsu, China

**Keywords:** post-stroke depression, rehabilitation, VOSviewer, Citespace, bibliometric analysis

## Abstract

**Background:**

Post-stroke depression (PSD) is a common complication of stroke and is associated with stroke prognosis. Rehabilitation plays an essential role in the comprehensive treatment of PSD. However, there are few bibliometric analyses of studies on PSD rehabilitation. This study aimed to comprehensively sort out the network of PSD rehabilitation through bibliometric analyses, analyze the research trends, focus on the hotspots related to PSD rehabilitation, and provide new research perspectives and guidance for future studies.

**Methods:**

The Web of Science Core Collection (WoSCC) database was searched for studies about depression rehabilitation after a stroke. The search covered the period from January 1, 2003, to October 31, 2024. We analyzed countries, institutions, journals, authors and keywords using CiteSpace and VOSviewer software to create visualizations and perform a bibliometric analysis.

**Results:**

A total of 2,227 papers were analyzed, with an increasing trend in the number of papers published each year. The United States had the highest number of published articles (458 publications), and Maastricht University and Utrecht University were the most published institutions (56 articles). Archives of Physical Medicine and Rehabilitation is the journal with the most cited publications (5,913 citations). Johanna M. A. is the most prolific author (24 publications).

**Conclusion:**

Using bibliometric methods, relevant studies on PSD rehabilitation were reviewed. The hotspots of future research on PSD rehabilitation will center on the brain plasticity mechanism of PSD rehabilitation, PSD assessment, and new techniques of PSD rehabilitation. This article provides systematic information to support and guide future research in this area.

## 1 Introduction

Stroke is a severe cerebrovascular disease with high morbidity, mortality and disability rates (1). It not only leads to impairment of motor, speech and cognitive functions but also adversely affects the psychological condition of patients (2). Post-stroke depression (PSD) is a prevalent complication that significantly impacts recovery and quality of life (3). In addition to stroke symptoms, it mainly manifests as a series of depressive symptoms with low mood and loss of interest, often accompanied by corresponding somatic symptoms (4). PSD is a clinical syndrome with a high incidence, with studies reporting a 38.1% prevalence among stroke patients 1 month after onset (5). Stroke survivors frequently experience emotional challenges, as highlighted in the multicenter DESTRO study by Paolucci et al., which involved 53 centers and 1,064 consecutive stroke patients. In this study, PSD was found in 36% of patients, with depression occurring within 3 months of stroke in about 80% of cases. Most cases were mild rather than major depression and identified risk factors included a history of depression, severe disability, prior stroke, and female gender. Patient autonomy and quality of life were severely affected (6). The prevention, early diagnosis, and early treatment of PSD is urgent, as it affects patient recovery and life expectancy and increases the risk of disability and death (7).

Patients with a confirmed clinical diagnosis of PSD are typically treated clinically with standardized antidepressant medication, but the evidence base lacks large randomized controlled trials in PSD populations, and the potential bleeding risks of the medication may be detrimental to stroke rehabilitation (8). Research indicates that patients with PSD are at a higher risk of mortality and disability problems than non-depressed stroke patients (9). Nonetheless, it is surprising that PSD is frequently neglected within the community, as deficiencies in treatment and diagnosis hinder patients from obtaining the necessary rehabilitation assistance (10). Moreover, many PSD patients experience social isolation due to persistent communication difficulties, often withdrawing from social interactions as a form of self-protection. Depression in post-stroke patients is particularly significant, as it profoundly affects various aspects of daily life (11). Depression reduces quality of life and impedes stroke recovery (12). One cause of PSD is damage to stroke-induced damage to specific brain regions. For example, patients who have had a lesion in the left frontal lobe or left basal ganglia often experience more depression than those with damage in other areas of the brain (13). Another potential cause of PSD is medication. Patients who experience hypertension after a stroke may be prescribed beta-blockers to reduce their blood pressure; however, they could face an increased risk of depression as a side effect (14). Although antidepressants are effective in treating PSD, their use is often limited by adverse drug reactions and poor patient compliance (15). With advancements of rehabilitation medicine, more and more medical practitioners are focusing on the rehabilitation of PSD.

Despite increasing scholars focusing on PSD rehabilitation, the research directions and hotspots in this field remain unclear. A comprehensive bibliometric analysis has yet to be conducted to systematically examine PSD rehabilitation. Bibliometric analysis quantitatively examines knowledge carriers using mathematical and statistical methods. This field merges mathematics, statistics, and bibliography, focusing on quantification (16). Bibliometrics, on the other hand, takes literature or literature-related media as the object of study and adopts mathematical, statistical, and different measurement methods to analyze quantitative relationship within the literature and explore the dynamic evolution of science and technology (17). VOSviewer and CiteSpace are the most commonly used tools for bibliometric analyses (18). In order to understand the overall landscape and development trends of PSD rehabilitation, this study statistically analyzes the published literature in this field. It aims to provide a comprehensive review of rehabilitation studies on PSD published from 2003 to the present, to explore the current status, research hotspots, and development trends in this field, and to identify emerging research directions. Additionally, this study provides scholars with intuitive information and guidance for future research.

## 2 Methods

### 2.1 Data source and collection

**Figure 1:**
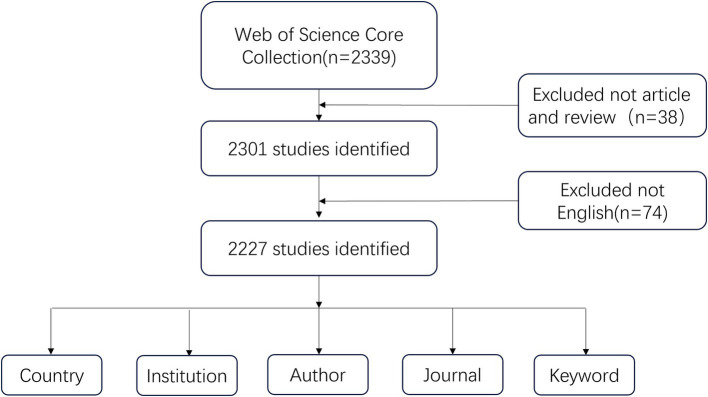
Flow chart of the research process.

This study selected Web of Science Core Collection (WoSCC) as the source database for data retrieval. We systematically searched all publications published between January 1, 2003, and October 31, 2024, to avoid bias due to database updates. The data retrieval strategy is as follows: ((TS = (“stroke” OR “cerebral ischemia” OR “cerebrovascular accident” OR “apoplexy” OR “encephalorrhagia” OR “cerebral hemorrhage” OR “brain vascular accident”) AND TS = (“depression”)) OR TS = (“post-stroke depression”)) AND TS = (rehabilitation). The publications were confined to articles and reviews, and a screening process was conducted on the titles and abstracts of all articles retrieved via the aforementioned search strategy. To ensure the accuracy of the bibliometric analyses, irrelevant materials such as book chapters, editorial content, and abstracts from conferences were excluded. In order to enhance the depth of the bibliometric content analysis, we have restricted the publication language to English. This limitation has resulted in a total of 2,227 original publications, encompassing both articles and reviews, that are presented in the English language. Figure 1 illustrates the process of selecting research literature. Each article includes a complete record of the country, institution, journal, author and keyword in text format exported from the WoSCC database.

### 2.2 Analysis methods

Comprehensive bibliometric analysis tools were employed to thoroughly investigate the research literature pertaining to depression rehabilitation in stroke patients. This analysis was conducted using the software programs VOSviewer version 1.6.20 and CiteSpace version 6.4.R1, allowing for a detailed exploration of trends, patterns, and critical insights in this crucial area of study. VOSviewer is a scientific measurement tool designed to generate and visualize co-occurrence networks. This study utilized VOSviewer to generate co-occurrence maps for countries, organizations, journals, authors, and keywords. In these networks, the size of each node visually represents its frequency or significance within the system. Larger nodes indicate a higher number or level of activity, while the lines connecting these nodes illustrate the collaborative relationships. These connections highlight how different entities interact and work together, forming a dynamic web of cooperation and exchange (19). CiteSpace integrates bibliometric analyses with systematic mapping to capture hotspots and research trends using data mining algorithms and visual analysis methods (20). In this study, CiteSpace was used to analyze a dual graph overlay of journals and clustered views of keywords. Dual graph overlay analysis reveals differences and associations between two network layers by overlaying them together. In CiteSpace, this feature is handy for journal analysis and can help researchers compare and analyze network structures under different topics or periods. By overlaying the two graphs, researchers can observe the changes and evolution of the nodes in the network, discover the differences in the network structure under different topics or periods, and identify the association patterns between the nodes (21). For keyword analysis, the log-likelihood ratio (LLR) strategy is employed to generate clusters of keywords by selecting the “Keywords” type.

## 3 Results

### 3.1 Analysis of publication outputs

**Figure 2:**
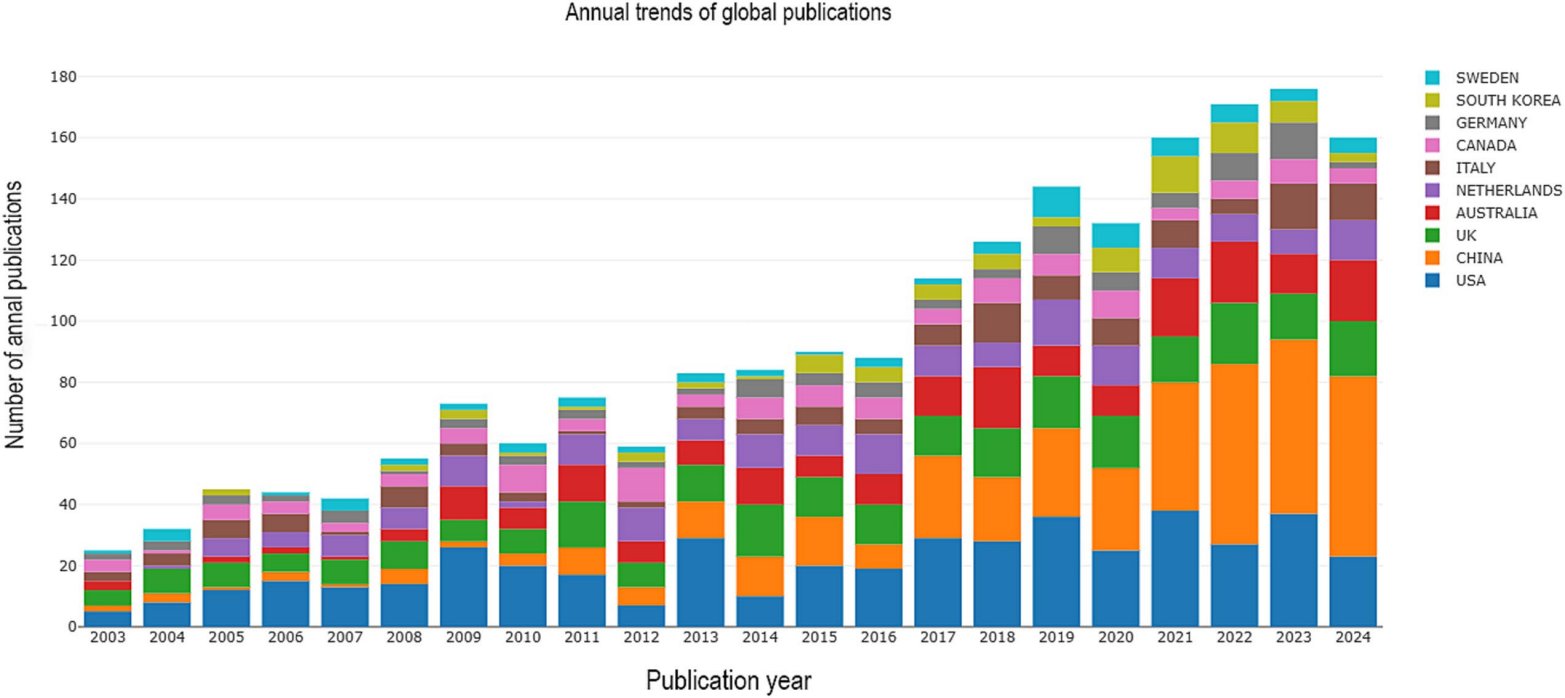
The distribution of annual publications on PSD rehabilitation from 2003 to 2024.

The analysis of publication output identified a total of 2,227 papers, which included 1,896 research articles and 331 review articles. These publications span the period from 2003 to 2024, reflecting a significant body of work in the field during these two decades. Figure 2 shows that the number of publications in this field in China was still low before 2010, started to increase in 2010, was low in 2016, and then rose steadily, with an overall trend of continuous and steady growth worldwide. In Europe, countries such as the United Kingdom, the Netherlands and Germany have long been at the forefront of PSD rehabilitation. The United States dominates this field of research. Low-income countries, such as North America, have fewer relevant publication outputs. The rehabilitation of stroke patients dealing with depression has increasingly become a focal point in recent years, highlighting the urgent need for comprehensive support.

### 3.2 Analysis of countries and institutions

**Figure 3:**
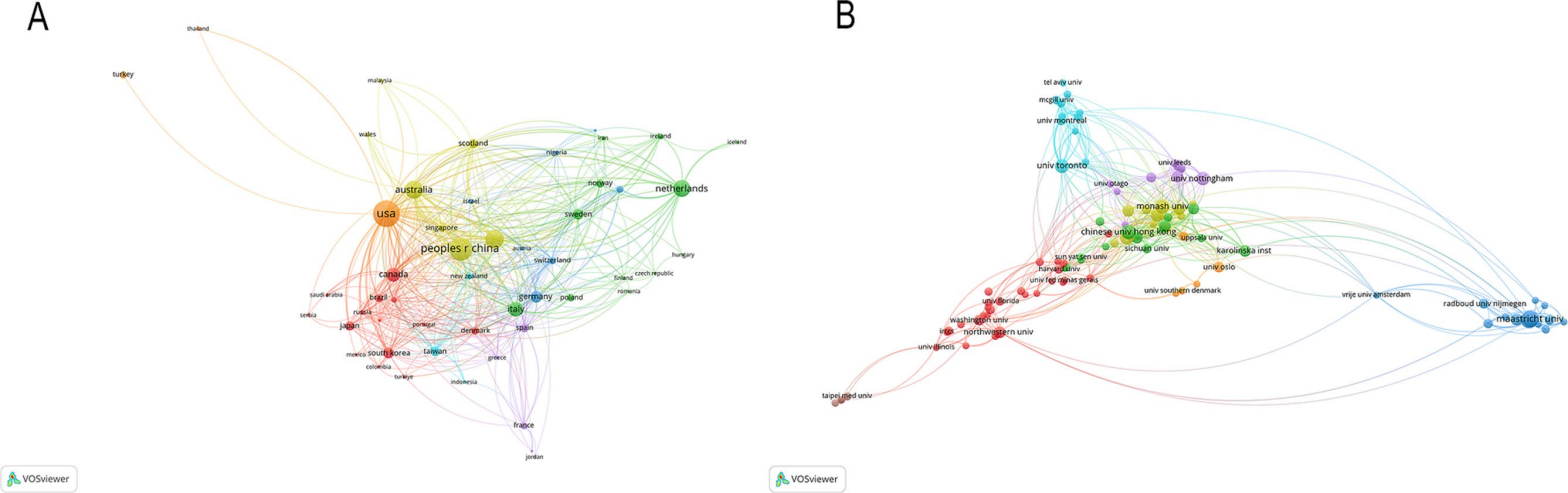
**(A)** Country co-occurrence map. **(B)** Institution co-occurrence map.

According to the authors’ addresses, PSD rehabilitation studies were conducted in 381 countries and 7,916 institutions. Table 1 presents the top 10 countries and institutions based on the number of publications they have produced. The United States had the highest number of publications in this field (458 articles, 14,991 citations), followed by China (353 articles, 6,021 citations), England (225 articles, 10,309 citations), Australia (211 articles, 7,999 citations) and Netherlands (186 articles, 6,609 citations). Figure 3A shows the cooperation network between the countries, where the size of each node indicates the number of publications published in each country. The institutions with the most publications are Maastricht University (56 articles, 1,375 citations) and Utrecht University (56 articles, 1,448 citations), followed by Monash University (44 articles, 879 citations), Chinese University of Hong Kong (43 articles, 1,446 citations), University of Toronto (41 articles, 1,983 citations) and University of Melbourne (40 articles, 1,201 citations). Figure 3B shows the collaborative network of institutions, forming eight color clusters based on the intensity of collaboration. The networking with most institutions is in red.

**Table 1:** The top 10 countries and institutions in PSD rehabilitation research.

Rank	Country	Counts	Citations	Institution	Counts	Citations
1	USA	458	14,991	Maastricht University	56	1,375
2	China	353	6,021	Utrecht University	56	1,448
3	England	225	10,309	Monash University	44	879
4	Australia	211	7,999	Chinese University of Hong Kong	43	1,446
5	Netherlands	186	6,609	University of Toronto	41	1983
6	Italy	135	4,174	University of Melbourne	40	1,201
7	Canada	127	5,671	University of Nottingham	36	1,166
8	Germany	92	3,347	La Trobe University	33	1,127
9	South Korea	79	1,588	Karolinska Institute	29	624
10	Sweden	76	2095	University of London	29	2,293

### 3.3 Analysis of journals

**Figure 4:**
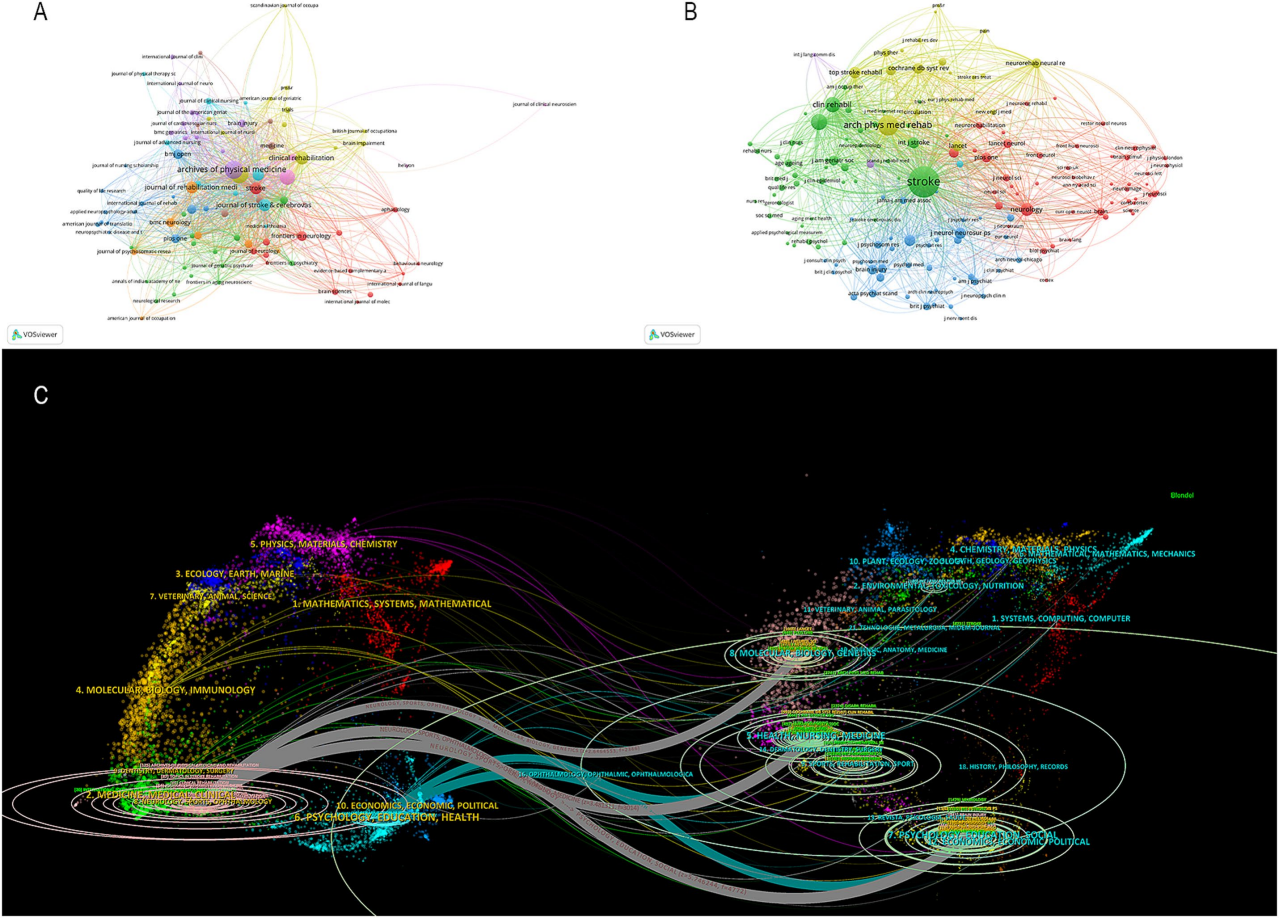
**(A)** Cited journal map. **(B)** Co-cited journals map. **(C)** Dual-map overlaps of journals in PSD rehabilitation.

As shown in Figure 4A, 493 journals analyzed published articles related to PSD rehabilitation. Based on the number of publications in Table 2, the top three journals were Archives of Physical Medicine and Rehabilitation (125 publications), Disability and Rehabilitation (122 publications), and Topics in Stroke Rehabilitation (87 publications). Stroke was the most co-cited journal (8,740 citations), followed by Archives of Physical Medicine and Rehabilitation (3,741 citations) and Disability and Rehabilitation (2,380 citations). Figure 4B illustrates the co-occurrence relationship among frequently cited journals. Figure 4C effectively illustrates the dual maps overlay of the journals, providing clear insights into their relationship. The map has two sections. The left side shows where cited journals are distributed, and the right side shows another distribution of cited journals. The ellipses’ lengths and widths on the map’s left show how many authors and publications there are. The lines connecting the left and right sides of the map indicate citation links that provide the complete context. Z-scores further highlight smoother trajectories, with thicker links indicating higher scores. We identified three main citation trajectories, with Neurology, Sports, and Ophthalmology cited significantly more often by Psychology, Education, and Social (Z = 5.75, *f* = 4,772); Health, Nursing, and Medicine (Z = 3.48, *f* = 3,014); and molecular, biology, genetics (Z = 2.65, *f* = 2,366).

**Table 2:** The top 10 authors and co-cited authors in PSD rehabilitation research.

Rank	Author	Counts	Co-author	Citations
1	Johanna M.	24	Hackett M.	659
2	Caroline M.	22	Robinson R.	534
3	Gerard M	17	Zigmond A.	331
4	Marcel W. M.	16	Duncan P.	300
5	Rocco Salvatore	15	Folstein M.	287
6	Majanka H.	15	Paolucci S.	278
7	Gert	14	Feigin V.	278
8	Julie	13	Ayerbe L.	263
9	Rosaria	12	Mahoney F.	246
10	Maree l.	12	Beck A.	201

### 3.4 Analysis of authors

**Figure 5:**
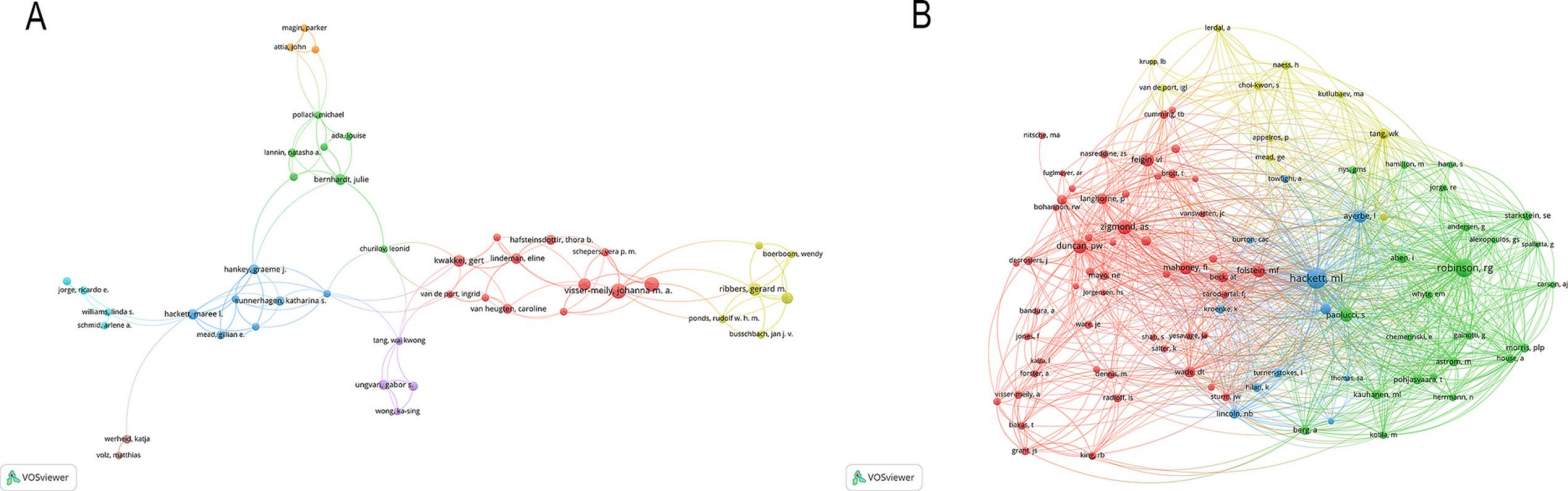
**(A)** Cited author map. **(B)** Co-cited author map.

A total of 10,217 authors have made valuable contributions to a wide range of research papers that focus on PSD rehabilitation. By examining the authorship of these publications, it is possible to identify notable scholars and influential research experts in the field by analyzing their academic publications, contributions to key conferences, and their impact on ongoing studies. The top 10 authors and co-cited authors in PSD rehabilitation research are listed in Table 3. This ranking is based on the number of publications each author has. Johanna M published the most articles (24 publications), followed by Caroline M (22 publications). Hackett M (659 citations) was the most cited author, followed by Robinson R. (534 citations) and Zigmond A. (331 citations). Figure 5 shows how authors collaborate with each other and with co-cited authors in the field. Circles represent the authors, and lines connect the circles to show their relationships. In Figure 5, a total of 152 authors, each with at least five publications, were grouped into 10 distinct clusters, and authors between clusters collaborated more closely. Research teams studying PSD rehabilitation should work together more across different regions in the future.

**Table 3:** The top 10 journals and co-cited journals in PSD rehabilitation research.

Rank	Journal	Counts	Co-cited journal	Citations
1	Archives of Physical Medicine and Rehabilitation	125	Stroke	8,740
2	Disability and Rehabilitation	122	Archives of Physical Medicine and Rehabilitation	3,741
3	Topics in Stroke Rehabilitation	87	Disability and Rehabilitation	2,380
4	Clinical Rehabilitation	65	Clinical Rehabilitation	2,114
5	Journal of Stroke & Cerebrovascular Diseases	59	Neurology	1,480
6	Journal of Rehabilitation Medicine	54	Topics in Stroke Rehabilitation	1,204
7	Stroke	53	Journal Of Neurology Neurosurgery And Psychiatry	1,122
8	Neuropsychological Rehabilitation	49	Cerebrovascular Diseases	1,118
9	Frontiers in Neurology	38	International Journal Of Stroke	1,109
10	BMJ Open	37	Cochrane Database of Systematic Reviews	1,009

### 3.5 Analysis of keywords

**Figure 6:**
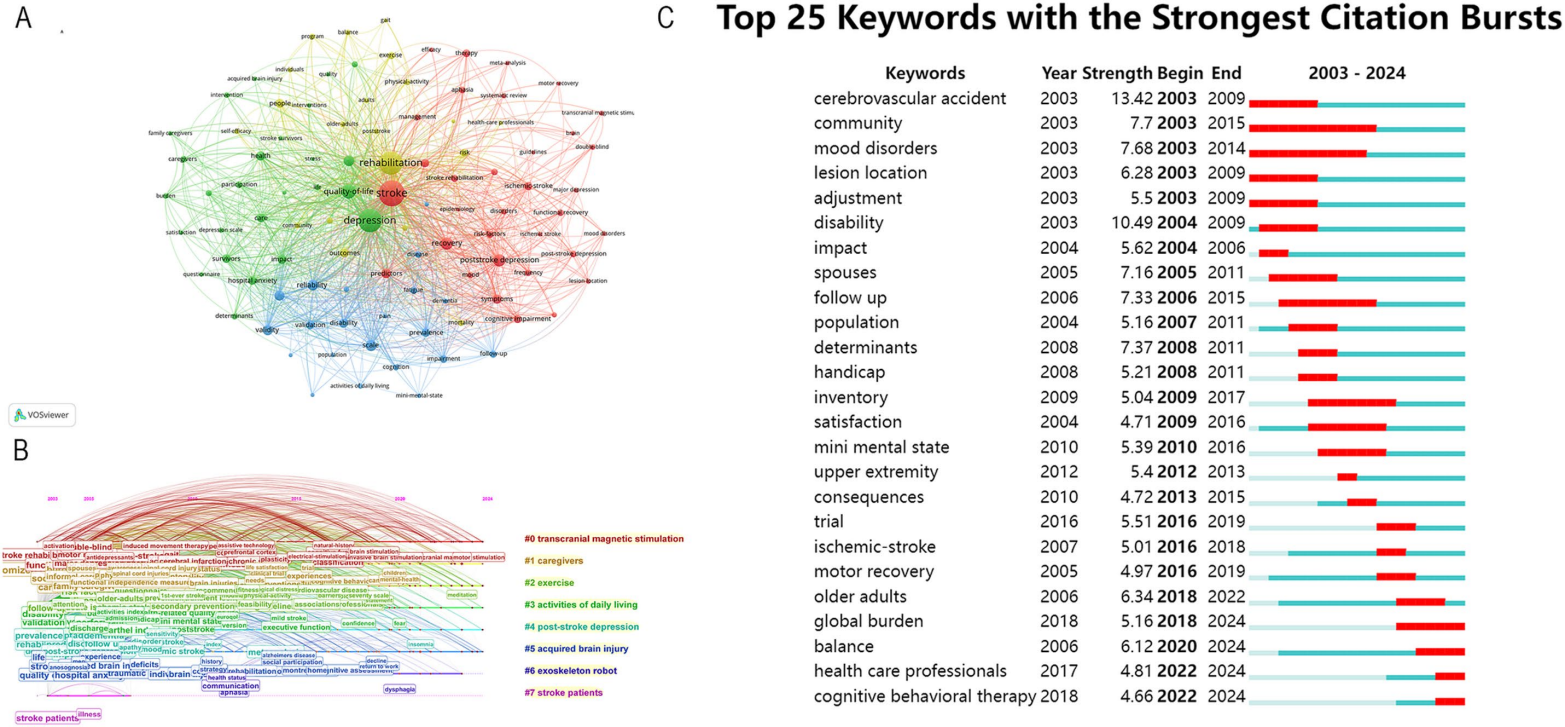
**(A)** Keyword co-occurrence map. **(B)** Timeline view of keywords. **(C)** Top 25 keywords with the strongest citation bursts from 2003 to 2024.

High-frequency keyword repetitions provide insights into current research topics and help predict future research trends. As illustrated in Figure 6A, the three most frequently occurring keywords are depression, rehabilitation, and quality of life. Other high-frequency keywords mainly include the severity and symptoms of PSD in PSD rehabilitation. The keywords can be classified into the eight clusters shown in Figure 6B based on the different types of keywords. Clusters #0, #2and #6 mainly describe that transcranial magnetic stimulation (TMS), exercise, and exoskeleton robot are widely used in PSD rehabilitation. Clusters #1 and #3 mostly show that caregivers, activities are also vital in PSD rehabilitation. The citation burst analysis is a good mapping that provides a report of hotspots and trends in a given year that are well analyzed for a specific research area. We utilized Citespace to identify the top 25 keywords with the strongest citation bursts, and the results are presented in Figure 6C. These keywords have been grouped into three periods according to the timing of their bursts: spanning from 2003 to 2009, 2010 to 2018, and 2018 to 2024. Among these keywords, cerebrovascular accident and disability had high burst intensity, and global burden, balance, health care professionals, and cognitive behavioral therapy were the most recent appearances. Keywords serve as indicators of recent research directions.

## 4 Discussion

### 4.1 Global trends of PSD rehabilitation research

This study analyzed 2,227 publications on PSD rehabilitation from 2003 to 2024, aiming to identify current research hotspots and trends. The results indicate a steady increase in annual publications. A temporary decline in 2016, possibly due to funding cycles and healthcare policy changes, did not disrupt the overall upward trend. The increase in PSD rehabilitation research publications in China after 2010 may be attributed to multiple factors, including policy support, international collaboration, advancements in research capacity, and growing societal demand. The findings suggest that more countries worldwide are now focusing on research about PSD rehabilitation.

Among the top 10 countries with the most publications in PSD rehabilitation research, nine are developed nations, with China being the sole developing country represented. Despite a late start in this field, China has shown rapid progress since 2010, with a significant rise in publications. It is also important to recognize the contribution of countries with low publication outputs, especially those in low and middle-income regions. These nations may face significant challenges in conducting research, such as limited access to resources, research infrastructure and expertise. However, their unique perspectives and experiences can offer valuable insights into localized PSD rehabilitation strategies, potentially fostering innovative and culturally adapted interventions. Further efforts are needed to support research in these areas and to ensure that their contributions are recognized. Despite increased global research activity, developing countries maintain fewer collaborative ties with developed nations in PSD rehabilitation, indicating a lack of international cooperation. This is also evidenced by the network of institutional collaborations in the above results; the institution co-occurrence map shows that institutions in developed countries still dominate the field of rehabilitation research in PSD. Notably, Maastricht University, Utrecht University, and Monash University rank among the most influential contributors. These findings suggest that the most prolific and authoritative institutions in PSD rehabilitation research are typically well-established universities with extensive academic resources in developed nations, highlighting a gap in educational exchange between developed and developing countries. The disparities can be attributed to two primary factors: First, research on PSD rehabilitation commenced earlier in developed countries. Second, insufficient attention and financial constraints in developing countries have hindered progress in this field, potentially contributing to the scarcity of high-quality research from these regions.

The rapid development of PSD research is closely linked to interdisciplinary collaboration across neuroscience, psychology, physical therapy, pharmacology, and social sciences. The dual graph overlay of journals also shows the importance of collaboration between multiple fields. For instance, developments in neuroimaging have facilitated precise mapping of depression-related neural circuits, such as prefrontal-limbic system connectivity disorders, aiding in the understanding of pathological mechanisms (22). Meanwhile, psychological theories such as the Cognitive Behavioral Model provide scientific guidance for targeted interventions (23). In terms of clinical interventions, studies integrating neuroplasticity biomarkers and behavioral therapies have shown that multimodal interventions are more effective in improving depressive symptoms than single-modality treatments, such as TMS or cognitive-behavioral therapy (CBT) alone (24). Additionally, exercise-induced upregulation of brain-derived neurotrophic factor (BDNF) has been shown to enhance antidepressant effects, reducing reliance on high-dose medications and optimizing the synergy between physical therapy and pharmacological interventions (25). Emerging research also highlights the pivotal role of social determinants, such as caregiver support and community involvement, in PSD rehabilitation (26). In countries with well-developed social welfare systems, integrated care models that combine clinical treatment with social rehabilitation have effectively reduced PSD incidence (27). Moving forward, PSD rehabilitation should further emphasize multidisciplinary collaboration, integrating neuromodulation, psychological interventions, exercise therapy, pharmacological treatments, and social support to develop individualized treatment plans. These efforts will enhance overall rehabilitation outcomes and improve the quality of life for patients.

### 4.2 Research hotspots

Keywords can help to understand research hotspots and future research directions. Therefore the following keyword-based analysis provides an opinion on the future research direction hotspots of PSD rehabilitation.

#### 4.2.1 Prediction and assessment of PSD

Evaluation is a crucial component of rehabilitation, and early identification and prediction of PSD are essential for preventing its progression. Timely identification of PSD is beneficial to patients’ physical and mental recovery (28). However, stroke is often accompanied by various neuropsychiatric symptoms, including apathy, anxiety, and fatigue, which overlap with PSD symptoms, making accurate diagnosis challenging (29). Currently, no specific tool for assessing PSD, and standard depression scales are primarily used to evaluate psychiatric symptoms of PSD. Such as the Patient Health Questionnaire (PHQ-9), Hamilton Depression Scale (HAMD), Beck Depression Inventory (BDI), and Hospital Anxiety and Depression Scale (HADS) (30). Future research should focus on the development of tools to assess PSD, such as blood markers (31). It has been shown that serum prealbumin is significantly higher in patients without PSD than in patients with PSD and that prealbumin is a significant predictor of PSD after adjusting for various confounding factors (32). A prospective cohort study identified fibrinogen as the primary predictive marker for PSD in men at 3 months post-stroke, while free T3, BDNF, and magnesium were key indicators in women (31). Additionally, some studies suggest that homocysteine may serve as a potential biochemical marker for depression in elderly stroke patients (33). Wen et al. propose that oxidative stress and inflammation after stroke contribute to PSD, providing new insights into its prediction and treatment of PSD (34).

#### 4.2.2 Repetitive transcranial magnetic stimulation for PSD

The #0 cluster is repetitive Transcranial Magnetic Stimulation (rTMS), an increasingly important treatment modality for PSD. rTMS is a painless and non-invasive physical therapy technique that employs electromagnetic induction to generate magnetic pulses by stimulation coils placed on specific areas of the scalp, which pass through the skull into the cerebral cortex, affecting areas related to mood and cognitive regulation to improve patients’ mood, sleep, and cognitive problems (35). Studies have shown that high-frequency rTMS (HF-rTMS) stimulation of the left dorsolateral prefrontal cortex (DLPFC) may have long-term antidepressant effects in patients with drug-resistant major depressive disorder (36). HF-rTMS of the left DLPFC has been reported to improve depressive symptoms in the subacute phase of subcortical ischemic stroke, with initial depressive symptoms at admission serving as a predictor of treatment outcome. Conversely, low-frequency rTMS (LF-rTMS) targeting the primary motor cortex (M1) did not improve depressive symptoms. In patients with mild-to-moderate PSD, rTMS may be the best option for early treatment (37). A randomized controlled trial by Li et al. demonstrated that rTMS of the left DLPFC was effective in improving cognitive impairment and depression in patients with PSD (38). A meta-analysis showed that applying high-frequency rTMS on the left DLPFC is an effective alternative for the treatment of PSD (39). However, there is no definitive consensus on the efficacy difference between low-frequency and high-frequency rTMS for treating PSD (40). Some studies have demonstrated similar efficacy. In contrast, others indicate differences in the effectiveness of various rTMS frequencies for treating PSD. Furthermore, research suggests that rTMS not only alleviates depressive symptoms in PSD patients but also sustains its efficacy beyond the treatment period (41). In the treatment of major depressive disorder, HF-rTMS has demonstrated significant antidepressant effects on the left DLPFC, while LF-rTMS may exert antidepressant effects on the right DLPFC. Some studies indicate no significant difference in antidepressant efficacy between right LF-rTMS and left HF-rTMS (42). However, bilateral rTMS stimulation of the DLPFC is not currently recommended, as its antidepressant effect may be primarily attributed to enhancing the efficacy of antidepressant medications (43). In summary, rTMS is a safe, efficient, painless, easy-to-use, and non-invasive physiotherapy technique that shows great potential in PSD treatment. With the deepening of research and the continuous advancement of technology, rTMS is expected to become one of the essential means of PSD treatment. Future research should focus on efficacy comparisons, long-term outcomes, combination therapies, and optimization of therapeutic parameters to further advance this field.

#### 4.2.3 Exercise for PSD

According to cluster #2, exercise has a significant positive effect on PSD (44). By choosing the right type and intensity of exercise, patients can gradually improve their mood, promote neuroplasticity, and enhance their social skills and quality of life (45). As research progresses, more and more researchers are focusing on the development of precision exercise prescriptions. This type of prescription creates a personalized exercise plan based on the patient’s specific situation, such as age, gender, and physical condition. Precision exercise prescriptions can improve the symptoms of PSD more effectively and enhance the rehabilitation effect (46). Traditional Chinese mind–body therapies have a significant impact on the improvement of psychological status. A 12-week period of personalized seated tai chi exercises developed for subacute stage stroke significantly improved patients’ depression (47). The results of the Meta-analysis recommended that stroke patients exercise less than 60 min per session, more than three times per week, and for a total of less than 180 min per week to achieve the best results against negative emotions (48). However, the number of randomized controlled trials of exercise to improve PSD is still low, and high-quality clinical studies should be conducted in the future to investigate the role of different exercise modalities. Cluster #5 Exoskeleton robots are wearable devices that can simulate the human body’s movement process and provide personalized rehabilitation training for patients (49). As a report shows that adding 8 weeks of exoskeleton robotics to conventional physical therapy can have a more positive impact on stroke patients (50). This positive impact may stem from the exoskeleton robot’s ability to increase patients’ confidence and motivation in rehabilitation, improve social skills and quality of life, and promote neuroplasticity (51). In the future, exoskeleton robots are expected to play a more significant role in stroke rehabilitation, bringing patients more hope for recovery and better quality of life (52). Meanwhile, further attention must be paid to the research hotspots on the long-term efficacy of exoskeleton robots in treating PSD.

#### 4.2.4 Acupuncture for PSD

The efficacy of acupuncture in treating PSD has gained increasing recognition among both patients and healthcare professionals. The proposed mechanisms underlying its therapeutic effects have expanded to include the regulation of neurotransmitters, reduction of neuronal damage, inhibiting hyperfunction in the hypothalamic–pituitary–adrenal axis, modulation of immune-inflammatory responses, and regulation of intestinal flora, among others (53, 54). Research indicates that the development of PSD is associated with neurobiological changes in the brain following brain tissue injury and that neurochemical dysregulation may be an important etiological factor (55). Acupuncture affects the release of critical central neurotransmitters such as monoamines, amino acids and peptides through the endogenous network and alleviating the physical symptoms of PSD (56). Furthermore, studies have demonstrated that acupuncture can enhance functional brain connectivity by increasing the fast wave power spectrum and decreasing the slow wave power spectrum, ultimately improving neurological function and alleviating depressive symptoms in PSD patients (57). Zhang et al. found that low-frequency (2 Hz) electroacupuncture stimulation of the Sishencong simultaneously improved PSD, potentially through the inhibition of activity in the right superior medial frontal lobe (58). In addition, acupuncture has been reported to positively affect other stroke-related symptoms, including physical function, speech and cognition, further enhancing its overall therapeutic efficacy on PSD (59). Moreover, acupuncture combined with music therapy (60), rTMS (61), and psychological interventions (62) has been shown to yield superior therapeutic outcomes compared to acupuncture alone. Given these findings, diversified combination therapy is expected to become a hotspot and trend for future research.

#### 4.2.5 Neuroplasticity and non-invasive brain stimulation

Neuroplasticity, a fundamental mechanism underlying functional recovery after stroke, has garnered significant attention in PSD rehabilitation research in recent years. It refers to the brain’s ability to adapt to injury through structural and functional reorganization, involving processes such as synaptic remodeling, neurogenesis, and neural network reconstruction (63). PSD patients frequently exhibit abnormalities in neural networks within the prefrontal cortex, limbic system, and basal ganglia, with weakened functional connectivity in these regions directly contributing to depressive symptoms. Consequently, enhancing neuroplasticity has become a key target in PSD rehabilitation. Non-invasive brain stimulation techniques—including rTMS, transcranial direct current stimulation (tDCS), and transcutaneous auricular vagus nerve stimulation (taVNS)—can effectively promote neuroplasticity by modulating cortical excitability and directly influencing brain regions involved in emotion regulation, such as DLPFC (64). For example, high-frequency rTMS (>5 Hz) enhances the excitability of the DLPFC, promotes glutamatergic neurotransmission and the release of brain-derived neurotrophic factor (BDNF), which improves the symptoms of depression and accelerates the recovery of cognitive function (65). Conversely, low-frequency rTMS (≤1 Hz) mitigates mood disorders by inhibiting hyperactivity in the right DLPFC and restoring interhemispheric balance (43). In addition, a randomized controlled trial by Liu et al. demonstrated that taVNS combined with conventional rehabilitation therapy could reduce HAMD scores by activating the vagal norepinephrine pathway and promoting neuroregeneration in the hippocampal region (66). taVNS significantly increased amygdala-dorsolateral prefrontal cortex connectivity, which was associated with a decrease in the severity of depression (67). The results of the Meta-analysis showed that taVNS was an effective and safe treatment for mild to moderate depression with efficacy comparable to antidepressants (68). However, due to the low to very low quality of existing evidence, further multicenter, double-blind, randomized controlled trials are necessary to provide high-quality data on taVNS efficacy across different types and severities of depression. Recent studies have also found that tDCS improves emotion regulation in PSD patients by modulating cortical excitability and cross-hemispheric balance, and its effect is dose-dependent with stimulation duration and current intensity (69). Furthermore, daily 40-min sessions of transcranial alternating current stimulation (tACS) at 77.5 Hz and 15 mA—administered via one frontal and two mastoid electrodes—have demonstrated efficacy in alleviating depressive symptoms (70). Although current research supports the effectiveness of non-invasive brain stimulation for PSD, most studies have been limited by small sample sizes. Future research should aim to expand sample sizes and investigate optimal stimulation parameters, potential synergistic effects with other interventions (e.g., virtual reality, exoskeleton robotics, artificial intelligence), and precise targeting strategies based on multimodal neuroimaging to maximize treatment efficacy. The study of specific related mechanisms is equally important.

### 4.3 Research trends and prospects

The research trends in PSD rehabilitation have become increasingly diversified and refined. With the advancements of neuroscience, rehabilitation medicine, and psychology, researchers have begun to explore the pathogenesis of PSD in greater depth and are committed to developing more effective and personalized rehabilitation treatments (71). On one front, researchers are actively searching for biological markers of PSD to provide more targeted therapy for patients through precision medicine. By utilizing neuroimaging (72), genetic testing (73), and neurotransmitter assays (74), they aim to elucidate the relationship between PSD and brain structure, function, and genetic variation, thus providing a scientific basis for early diagnosis and intervention of PSD (75). Concurrently, with the continuous advancement of rehabilitation technology, researchers have begun to focus on functional recovery and quality of life improvement for PSD patients. This includes not only addressing emotional distress but also restoring cognitive function, motor function, and social ability (76). To achieve these goals, researchers are exploring a variety of rehabilitation therapies, such as cognitive-behavioral therapy (23) and non-invasive brain stimulation (77), and attempting to combine these approaches to form an integrated rehabilitation treatment plan (78). Additionally, keyword frequency analyses indicate a growing research focus on family and social support in PSD rehabilitation. Researchers recognize that strong family and social networks play a crucial role in patient recovery (79). They are exploring how to provide patients with more comprehensive and continuous rehabilitation services through family-centered interventions and community-based rehabilitation programs (80).

In conclusion, the research trend of PSD rehabilitation is developing in a more in-depth, refined and comprehensive direction. Future research should focus on several key areas. First, to address existing gaps in understanding the neural mechanisms and neuroplasticity of PSD, multimodal imaging, neurophysiology, and molecular biology should be leveraged to explore the intrinsic relationship between neural remodeling and rehabilitation outcomes following brain injury. Second, given the limitations of existing PSD assessment tools in accurate diagnosis and prognosis, a comprehensive evaluation system integrating biomarkers, imaging indicators, and clinical symptoms should be developed to enable early identification and intervention. Third, a multidisciplinary intervention model combining traditional treatments with emerging rehabilitation technologies—such as rTMS, virtual reality, artificial intelligence, and robotic rehabilitation—should be further explored. Large-scale, multicenter randomized controlled trials with long-term follow-ups are needed to validate the sustained efficacy of these interventions. Lastly, fostering interdisciplinary collaboration and international cooperation among fields such as neurology, rehabilitation medicine, psychology, and data science will provide new perspectives and platforms for elucidating the pathogenesis of PSD and optimizing rehabilitation strategies.

## 5 Limitations

This study has certain limitations. First, due to software constraints, we analyzed only publications from SCIE and SSCI in WoSCC, which may have led to the omission of some studies. Second, only English-language articles and reviews were included, potentially excluding valuable research published in other languages. In addition, due to some recent publications and low citation counts, a few recently published high-quality papers may not have been adequately considered. Finally, this analysis focused on bibliometric data global research trends, co-occurrence, and keywords related to PSD, resulting in limited exploration of PSD interventions. Future research should integrate both quantitative and qualitative approaches to address the inherent limitations of bibliometric studies.

## 6 Conclusion

A bibliometric analysis of the literature related to PSD rehabilitation in recent years reveals a trend of diversification and refinement of research in this field. In conclusion, future research should focus on elucidating the mechanisms underlying PSD rehabilitation, improving the prediction, identification, and precise assessment of PSD, and exploring emerging technologies such as non-invasive brain stimulation. Emphasis should also be placed on the development of large-sample, high-quality clinical studies to advance the field. This will enhance functional recovery and improved quality of life and is expected to provide a more comprehensive and effective rehabilitation program for patients.

## Data Availability

The datasets presented in this study can be found in online repositories. The names of the repository/repositories and accession number(s) can be found in the article/supplementary material.
